# Supplementary material for: Effect of general anesthesia and controlled mechanical ventilation on pulmonary ventilation distribution assessed by electrical impedance tomography in healthy children
Source: PLoS One. 2023 Mar 16;18(3):e0283039. doi: 10.1371/journal.pone.0283039 (PMC10019725; doi:10.1371/journal.pone.0283039)
Supplement: Attachment — Submitted filename: Response to Reviewers.docx [file pone.0283039.s002.docx]

**To the editor:**

Dear editor,

We would like to request the inclusion of an colaborator who participated in all stages of the study and unfortunately, we forgot to include at the time of submission: Caio C.A. Morais (Affiliation: Divisão de Pneumologia, Departamento de Cardiologia – Instituto do Coração (INCOR) Hospital das Clínicas, HCFMUSP, Faculdade de Medicina, Universidade de São Paulo, São Paulo, SP, BR).

**Reviewers' Comments to Authors:**

Reviewer #1: The manuscript is well written and easy to understand. The study was an observational clinical study with simple and clear objectives. The authors report their findings about the development of atelectasis in the dorsal area in mechanically ventilated patients with normal lungs who underwent nonthoracic surgery. They found that ventilation shifted from posterior to anterior region in the patients younger than 5 years as often observed in adult patients.

We appreciate the reviewers' time and effort dedicated to our manuscript, their comments and suggestions have certainly helped us improve our work. Below is a point-by-point answer to all the questions raised by the reviewers.

**(1) The statistical analysis section is not clear. Explain the methods more clearly with citations. Rationales for the chosen statistical methods would be helpful. For example, it is difficult to understand the following expressions:
- “models of generalized estimation equations with gamma or Poisson distribution and logarithmic link function”
- “mixed linear models with identity or logarithmic link functions according to the best fit, considering the dependence between the various measurements performed at each evaluation time and between the moments evaluated in the same patient”**

**RESPONSE**: We appreciate the suggestion given by the reviewer. We reviewed the text and some details of the statistical analysis. We took the opportunity to make some changes to the figures to make our results clearer for the reader. The explanation for the statistical analysis performed is detailed below, however, in the text of the manuscript we left it more succinct to make it clearer for the readers.

In order to compare ventilation parameters and Delta Z measurements between the study periods, we need to consider the dependence between the different measurements performed in the same period and between the different periods for the same child. The ventilation parameters (Table 2) show little or no variation in the same period (epochs), so to compare the mean of these parameters between the different periods we used generalized estimation equation (GEE) models. Delta Z measurements showed great variability between measurements of the same period and for this reason generalized mixed models were adjusted to make it possible to model the correlation structure of the random effect (patient) and also of the repeated factor (epochs).

As requested by the reviewer, a reference was inserted.

Reference: Faraway JJ. Extending the linear Model with R: Generalized Linear, Mixed Effects and Nonparametric Regression Models, Boca Raton: Chapman & Hall/CRC, 2006.

**TEXT:** “*The data were described as absolute and relative frequencies for the categorical variables and as medians and quartiles, in addition to minimum and maximum values for the numerical variables. The means of the mechanical ventilation parameters were obtained by fitting models of generalized estimation equations. Changes in global and regional ventilation were tested with linear mixed models, accounting for the repeated measurements with a random intercept. In addition, models were adjusted with subjects nested within time (induction-SB, MV-5min, MV-30min, MV-late, recovery-SB), considering the multiple measurements (breaths) from the same subject at each time point. The results of the models are presented as the mean values estimated by the models with corresponding 95% confidence intervals and p values (17). The analyses were performed with the R statistical software (R Foundation for Statistical Computing, Vienna, Austria) considering a significance level of 5%.”*

**MANUSCRIPT LOCATION**: Section Methods, page 09. We modified figures 2 and 3 as well as their legends.

**(2) Please provide explanations for greater ventilation in the posterior region during SB before anesthesia.**

**RESPONSE**: We appreciate the reviewer's inquiry and the opportunity to respond.

The literature has a very solid physiological foundation regarding regional differences in ventilation. During active inspiration, the dorsal part of the diaphragm has a greater range of incursion. In this region, the muscle fibers are more elongated, therefore, they can develop more strength (or shortening of the fibers) during inspiration. With greater amplitude of incursion, the variation of pleural pressure is greater, leading to greater ventilation of the posterior region. However, as soon as the effect of inhalation anesthesia and muscle relax take, ventilation is shifted toward the nondependent lung. One possible explanation is that during paralysis and in supine position, the anterior chest wall is more readily expanded as the posterior chest wall is splinted by the surface the patient is lying on and the cranial movement of the anterior diaphragm. During anesthesia of adult patients, the shape of the chest changes with a reduction in the cross-sectional area of the ribcage.

We have inserted a new reference in the manuscript and a new paragraph in the discussion to make the explanation more consistent.

***TEXT:*** *“During spontaneous breathing, diaphragmatic contraction has a strong influence on the direction of ventilation. The posterior portion of the dome presents greater amplitude during the incursion and, therefore, greater pressure variation in the posterior region of the thorax. During active inspiration, the dorsal part of the diaphragm has a greater range of incursion. In this region, the muscle fibers are more elongated, therefore, they can develop more strength during inspiration. With greater amplitude of incursion, the variation of pleural pressure is greater, leading to greater ventilation of the posterior region. However, as soon as the muscle relax take, ventilation is shifted toward anterior region. That because is during muscle relax and in supine position, the anterior chest wall is more readily expanded as the posterior chest wall is splinted by the surface the patient is lying on and the cranial movement of the anterior diaphragm. During anesthesia of adult patients, the shape of the chest changes with a reduction in the cross-sectional area of the ribcage.”*

**REFERENCES**

*Froese AB. Anesthesia-paralysis and the diaphragm: in pursuit of an elusive muscle. Anesthesiology. 1989 Jun;70(6):887-90. PMID: 2729628.*

*Reber A, Nylund U, Hedenstierna G. Position and shape of the diaphragm: Implications for atelectasis formation. Anaesthesia. 1998;53(11):1054–61.*

*Hedenstierna G, Lichtwarch-Aschoff M. Interfacing spontaneous breathing and mechanical ventilation. New insights. Minerva Anestesiol. 2006;72(4):183–98.*

*Wahba RWM. Perioperative functional residual capacity. Can J Anaesth. 1991;38(3):384–400.*

**MANUSCRIPT LOCATION**: Section Discussion, page 13

**(3) Please provide more explanations about the increased Delta Z during CMV. What was the mode of MV?**

We thank the reviewer for the question. The ventilatory modality used in all patients was pressure controlled and it is worth mentioning that, as this was an observational study, there was no control over the ventilatory parameters used by the anesthesiologists. In our study, as described in our discussion, there was an increase in Delta Z after starting positive pressure ventilation compared to spontaneous breathing at the time of anesthetic induction. The literature corroborates our findings and shows that, compared to spontaneous breathing, the use of positive inspiratory pressures increased Delta Z by up to 50%.

Radke OC, Schneider T, Vogel E, Koch T. Effect of trigger sensitivity on redistribution of ventilation during pressure support ventilation detected by electrical impedance tomography. Anesthesiol Pain Med. 2015 Aug 1;5(4).

Humphreys S, Pham TMT, Stocker C, Schibler A. The effect of induction of anesthesia and intubation on end-expiratory lung level and regional ventilation distribution in cardiac children. Paediatr Anaesth. 2011 Aug;21(8):887–93.

**TEXT:** *“The analysis of the impedance variation, Delta Z, showed an increase in whole ventilation with the beginning of mechanical ventilation, a fact previously described once the use of positive inspiratory pressure results in higher tidal volumes compared to spontaneous breathing (27-28). Humphreys et al evaluating children undergoing cardiac surgery showed that the delta z almost doubles with the start of mechanical ventilation when compared to spontaneous breathing (28).”*

**MANUSCRIPT LOCATION**: Section Discussion, page 15

**(4) Please provide more explanations about the still-increased Delta Z during SB after surgery.**

During the period of controlled mechanical ventilation, 95% of our patients received opioids (fentanyl). Radke, in his study that compared the distribution of ventilation ~~in~~ during spontaneous breathing and ~~in~~ throughout mechanical ventilation, points out that the use of opioids for reducing the respiratory rate (RR)can influence the tidal volume (Vt). So, for maintaining a similar minute volume, a diminished RR would determine a higher Vt.

We speculate that the residual fentanyl effect during recovery time might relate to Delta Z increment in comparison to the induction time. However, no statistical significance was observed between the induction-SB and recovery-SB epochs.

**Journal Requirements:**

When submitting your revision, we need you to address these additional requirements.

1. Please ensure that your manuscript meets PLOS ONE's style requirements, including those for file naming.

**RESPONSE:** All text has been proofread and formatted according to the PlosOne’s style.

2. You indicated that you had ethical approval for your study. In your Methods section, please ensure you have also stated whether you obtained consent from parents or guardians of the minors included in the study or whether the research ethics committee or IRB specifically waived the need for their consent

**RESPONSE:** We acknowledge for this and we have inserted in the “Methods section” the statement “An informed consent form was obtained from each child’s parents.”

3. In your Data Availability statement, you have not specified where the minimal data set underlying the results described in your manuscript can be found. PLOS defines a study's minimal data set as the underlying data used to reach the conclusions drawn in the manuscript and any additional data required to replicate the reported study findings in their entirety. All PLOS journals require that the minimal data set be made fully available. For more information about our data policy, please see http://journals.plos.org/plosone/s/data-availability.

**RESPONSE:** We recognize this would be appropriate but unfortunately in Brazil we have a big restriction on data availability due to the data protection law and our institution does not have any registration with specific platforms for data deposit.

4. We note that the grant information you provided in the ‘Funding Information’ and ‘Financial Disclosure’ sections do not match.

When you resubmit, please ensure that you provide the correct grant numbers for the awards you received for your study in the ‘Funding Information’ section

**RESPONSE:** We reinforce that no funding or scholarship was granted for this study, but we will review the data in the ‘Funding Information’ section
