# Supplementary material for: Effect of general anesthesia and controlled mechanical ventilation on pulmonary ventilation distribution assessed by electrical impedance tomography in healthy children
Source: PLoS One. 2023 Mar 16;18(3):e0283039. doi: 10.1371/journal.pone.0283039 (PMC10019725; doi:10.1371/journal.pone.0283039)
Supplement: Attachment — Submitted filename: Response to Reviewers_.docx [file pone.0283039.s003.docx]

**To the editor:**

Dear editor,

Although our institution does not have any registration on specific platforms for data deposit, we are sharing our data as Supporting Information files. We appreciate the opportunity to be able to provide them in this way.
