# Supplementary material for: Effect of general anesthesia and controlled mechanical ventilation on pulmonary ventilation distribution assessed by electrical impedance tomography in healthy children
Source: PLoS One. 2023 Mar 16;18(3):e0283039. doi: 10.1371/journal.pone.0283039 (PMC10019725; doi:10.1371/journal.pone.0283039)
Supplement: Attachment — Submitted filename: Response to Reviewers.docx [file pone.0283039.s004.docx]

Reviewers' Comments to Authors:

We appreciate the reviewers' time and effort dedicated to our manuscript, their comments and suggestions have certainly helped us to improve our work. Below is a point-by-point answer to all the questions raised by the reviewers.

Reviewer #3: In this manuscript, Nascimento et al. describe the effect of general anesthesia on pulmonary ventilation distribution in children. The study was performed by experts in the field, was well conducted and is well written. However, the reviewer has some major concerns:

- What ventilatory mode was applied during the different epochs?

**RESPONSE:** We appreciate the reviewer's question. The ventilatory mode applied during all studied periods of mechanical ventilation was pressure controlled ventilation. We inserted a phrase in the “Results” section highlighting this aspect.

**TEXT:**  *Pressure Controlled ventilatory modality was used in all patients and no patient triggered cycle was detected during the analyzed periods.*

**MANUSCRIPT LOCATION**: Section “Results”, page 10.

- Were the patients allowed to breathe spontaneously during the different epochs?

**RESPONSE:** No, they were not allowed to spontaneously ventilate during the study period. As previously mentioned, all patients remained on controlled ventilation and did not have spontaneous cycles.

**TEXT:** *Pressure Controlled ventilatory modality was used in all patients and no patient triggered cycle was detected during the analyzed periods.*

**MANUSCRIPT LOCATION**: Section “Results”, page 10.

- Please provide (static) compliance during all epochs. I suggest that you will find significant changes. If this is not the cause, the amount of atelectasis may not be that great.

**RESPONSE:** We appreciate the reviewer's comments and compliance was obtained, and the values were entered in Table 2. There was no significant difference in compliance results when comparing the 3 periods of mechanical ventilation.

**TEXT:** The delta pressure (Pins - PEEP), VT adjusted for patient weight (in mL/kg) and compliance (mL/cmH2O) were calculated.

**MANUSCRIPT LOCATION**: Section “Methods”, page 08 and Section “Results”, table 2.

- Could you please provide an EIT analysis (Figure) using the center of ventilation? Maybe the reader can then decide which description is more intuitive.

**RESPONSE:** We appreciate the reviewer's comment. The figure with the CoV values was created and designate Figure 3. We inserted a sentence in methods.

**TEXT:** *The distribution of ventilation in percentage was presented as dorsal ventilation fraction, which was calculated by the ratio of the Delta Z of the posterior region by the global Delta Z multiplied by 100 and by center of ventilation (CoV) that is defined based on the center of gravity or center of mass and its value is given automatically by the EIT (17). (Methods)*

*When ventilation distribution was evaluated by CoV the results were like those found by the dorsal ventilation fraction. There was redistribution from the posterior to the anterior region observed with the onset of the MV (posterior ventilation went from 51% (IC95%: 49-53%) to 47% (IC95%:46-49%) (p<0.001). With the restoration of spontaneous breathing, ventilation distribution returned towards the normal pattern with predominant ventilation in the dorsal regions (posterior ventilation went from 48% (IC95%: 47-50%) to 49% (IC95%:47-51%), although a small difference persisted as compared to the induction-SB period but without significance (Fig 3). (Results)*

*Figure 3: Distribution of pulmonary ventilation assessed by center of ventilation (CoV) presented over the five time points: induction-SB, MV-5min, MV-30min, MV-late and recovery-SB. The CoV is a parameter that quantifies the distribution of ventilation. When we have a value of 50 it means that ventilation is equally distributed between the anterior and posterior regions of the thorax. Higher numbers indicate a shift towards the dorsal region, and lower numbers indicate a shift towards the ventral region. Significant variations were observed in the ventilation distribution measures demonstrated in percentage when compared MV-5min, MV-30min and MV-late to induction-SB.*

**MANUSCRIPT LOCATION**: Section “Methods”, page 08; Section “Results”, page 12 and figure legends.

- How would you change the ventilatory settings according to EIT measurements in respect of avoiding atelectasis, alveolar cycling and alveolar overdistension?

**RESPONSE:** The EIT presents several resources for monitoring and adequacy of mechanical ventilation. Among the most used EIT tools at the bedside we emphasize the ventilation distribution map (given in percentage), and the Delta Z, which has a strong correlation with tidal volume (Vt).

Checking alveolar cycling can be done also by the color scale automatically performed by the EIT apparel, where dark blue color relates to low Vt and lighter blue to white relates to high tidal volumes. This is done in real-time by the device, considering the relative Delta Z variation at each cycle.

To perform an EIT ventilation evaluation it is possible to elect the regions of interest (ROIs), such as anterior and posterior, in the vertical axis, for instance. However, a caution for this interpretation includes knowledge of how the behavior of a healthy lung is when exposed to mechanical ventilation and this was the main objective of this study.

Once we know that the distribution of anterior-posterior ventilation of healthy lung children on mechanical ventilation is quite similar, about 50% of Vt directed to the anterior and posterior ROIs, we can target this distribution when we adjust the ventilatory parameters at the bedside.

But, for instance if we observe a posterior ventilation distribution lower than 50% of the Vt and progressively decreasing, this suggests an ongoing collapse in this region, and then PEEP rise might be a good strategy. On the other hand, if we observe a posterior ventilation distribution above 50% with the child on mechanical ventilation, we can interpret that the anterior region is hyperinflated, since the non-dependent lung is more prone to earlier overdistension, and the adjustment of parameters such as PEEP reduction or tidal volume adjustment is necessary.

In addition, observation of Delta Z during ventilation provides information on Vt and regional ventilation. We may be ventilating with the theoretical concept of protective ventilation, however, when we look at regional ventilation, there are regions that are receiving much higher volumes than others when the lung aeration is not adequately titrated.

We consider that EIT allows an additional and different layer of monitoring, making ventilation safer and more effective.

With those concepts in mind, EIT will help tailoring ventilatory parameters at the bedside in a unique approach, likely minimizing atelectrauma and volutrauma, guiding when titrating ventilatory parameters at the bedside, monitoring patient decubitus changes and recruitment maneuvers.

We have inserted a sentence in the discussion section to make it clearer for the reader.

**TEXT:** *Once we know that in children with healthy lungs the anterior-posterior ventilation distribution during mechanical ventilation is quite similar, we might titrate the ventilatory parameters to maintain this distribution pattern. In accordance to this concept, if posterior zones ventilation is going below 50%, collapse might be present and then PEEP increase and FRC gain shall be the target. On the other hand, if posterior ventilation distribution is above 50%, anterior hyperinflation is expected and parameters adjustments such as PEEP reduction or tidal volume adjustment are likely necessary.*

**MANUSCRIPT LOCATION**: Section “Discussion”, page 15.

Minor concerns:

- Table 2: Please define all abbreviations. What do you mean with irpm? Please include tidal volume / predicted body weight

**RESPONSE:** All abbreviations have been inserted and the respiration rate unit has been adjusted. Tidal volume by weight was already in the table.

**MANUSCRIPT LOCATION**: Table 2.

- How can you assure that you included only lung-healthy patients?

**RESPONSE:** Children were eligible for the study presenting no clinical symptoms that would result in impact over the lungs, and only after evaluation by the anesthesiologist. In the anesthetist's evaluation, the parents were asked about a possible history of prematurity, prior or recent episodes of wheezing and about other chronic diseases such as cystic fibrosis. Thus, it was possible to admit that all included children did not present pulmonary diseases.

- Title: Please use the term “general anaesthesia”

**RESPONSE:** The term “general anesthesia” has been included in the title

**TEXT:** *Effect of general anesthesia and controlled mechanical ventilation on pulmonary ventilation distribution assessed by electrical impedance tomography in healthy children.*

**MANUSCRIPT LOCATION**: Title

- I suggest using measurement periods may be more appropriate than epochs

**RESPONSE:** All text has been revised and all terms "epochs" have been replaced by "periods"

**MANUSCRIPT LOCATION**: All text

- What does the reader really learn from Figure 2?

**RESPONSE:** The Figure 2 shows the distribution of pulmonary ventilation assessed by the dorsal ventilation fraction in the anterior and posterior regions presented. It Shows that there was a redistribution of ventilation from the posterior to the anterior region with the onset of mechanical ventilation and with the restoration of spontaneous breathing, ventilation distribution returned towards the normal pattern with predominant ventilation in the dorsal regions. When we use the EIT to evaluate the distribution of pulmonary ventilation, we expect to find a more homogeneous ventilation, that is, distribution of anteroposterior ventilation close to 50% anterior and 50% posterior.

**TEXT:** *There was a redistribution of ventilation from the posterior to the anterior region with the onset of MV (posterior ventilation went from 54% (IC95%: 49-60%) to 49% (IC95%:44-53%) (p = 0.002) and remained stable over the whole ventilation period (Fig 2). With the restoration of spontaneous breathing, ventilation distribution returned towards the normal pattern with predominant ventilation in the dorsal regions (posterior ventilation went from 51% (IC95%: 42-61%) to 56% (IC95%:51-62%) (p = 0.02), although a small difference persisted as compared to the induction-SB period but without significance (Fig 2).*

**MANUSCRIPT LOCATION**: Section “Results”, page 11.
