# Supplementary material for: Effect of general anesthesia and controlled mechanical ventilation on pulmonary ventilation distribution assessed by electrical impedance tomography in healthy children
Source: PLoS One. 2023 Mar 16;18(3):e0283039. doi: 10.1371/journal.pone.0283039 (PMC10019725; doi:10.1371/journal.pone.0283039)
Supplement: Attachment — Submitted filename: Response to Reviewers.docx [file pone.0283039.s005.docx]

Reviewers' Comments to Authors:

We appreciate the reviewers' time and effort dedicated to our manuscript, their comments and suggestions have certainly helped us to improve our work. Below is a point-by-point answer to all the questions raised by the reviewers.

Reviewer #4: In this manuscript, effects of general anesthesia on pulmonary ventilation distribution in children are examined. The study was performed by EIT. The study was by experts in the field, was well conducted and is well written. The previous comments and changes appear to have improved the quality, too. Some comments:

1. The study has been performed in 20 patients. Although this has been stated as a limitation, I wonder whether the study is "powered" enough to make some conclusion. My knowledge in statistics is not good enough to understand whether and how a comparison of 5 measurement times was possible in distribution of ventilation.

**RESPONSE:** We appreciate the reviewer's comments and this question has been shared with our team statistician. This issue was a concern of the authors when the study was idealized. However, this was the first study that would address the effect of anesthesia on ventilation distribution in the pediatric population. As there is no reference to pediatric values of the EIT data evaluated for sample calculation, it was decided to carry out a convenience sample. The problem in cases of small samples is that a type 2 or beta error may occur, which shows no significant difference when in fact there is such a difference. However, our results pointed to a significant difference in the distribution of ventilation (which was our main analysis variable), thus pointing to a low probability of type 2 error. We included a sentence at the end of the statistical analysis to make it clear for the reader.

Regarding the 5 evaluation moments, all moments were compared with the Induction-SB moment through linear mixed models. Having a large number of evaluations in a small sample is not really a problem. in addition, we considering the multiple measurements (breaths) from the same subject at each time pointe, this increases the power of the analysis as it enables a better fit of the model.

**TEXT** *As there is no reference to pediatric values of the EIT data evaluated for sample calculation, it was decided to carry out a convenience sample.*

*Changes in global, regional ventilation and CoV were tested with linear mixed models, accounting for the repeated measurements with a random intercept. In addition, models were adjusted with subjects nested within time (induction-SB, MV-5min, MV-30min, MV-late, recovery-SB), considering the multiple measurements (breaths) from the same subject at each time point. The results of the models are presented as the mean values estimated by the models with corresponding 95% confidence intervals and p values (18).*

**MANUSCRIPT LOCATION**: Section “Materials and methods”, page 9.

2. similarly, I would wonder whether different ventilation strategies would lead to different distributions of ventilations; but first of all, the strategy is more or less very similar (i.e. almost the same PEEP), and second, sample size is too low for any assumption.. But at least, has there been any recruitment manover in any of the patients in any time?

**RESPONSE:** We appreciate the reviewer's comment. The use of spontaneous breathing during mechanical ventilation has been discussed, in adults, as a strategy to prevent redistribution of ventilation in dependent zones, however, with controversial results. But the objective of our study was not to compare modalities and therefore, all our patients were ventilated in pressure-controlled mode without spontaneous cycles during mechanical ventilation. Despite our small sample size, our results show a significant difference in the distribution of ventilation with the use of controlled mechanical ventilation when compared with spontaneous breathing (without mechanical ventiltion) and this supports our conclusion. Inserted a sentence and 2 references in the discussion to complement the ventilatory strategy.

**TEXT** *The use of spontaneous breathing during mechanical ventilation has been discussed as a strategy to prevent the redistribution of ventilation in dependent zones, however, with controversial results (20,21).*

**MANUSCRIPT LOCATION**: Section “Discussion”, page 13 and References.

20.	Radke OC, Schneider T, Heller AR, Koch T. Spontaneous breathing during general anesthesia prevents the ventral redistribution of ventilation as detected by electrical impedance tomography: A randomized trial. Anesthesiology. 2012;116(6):1227–34.

21.	Putensen, Christian; Muders, Thomas; Varelmann, Dirk; Wrigge H. The impact of spontaneous breathing during mechanical ventilation. Curr Opin Crit Care. 2006;12(1):13–8

3. Difference adult- child: there is some classical info written in the discussion.. But i wonder : what are the differences in EIT between adult and child? can you discuss the previous study made in adults with this one in child? (I am aware that a "comparison" is not possible, but the reader would like to know a simple thing: is the change caudes by mechanical ventilaton less or more in children? at least, maybe some speculations)

**RESPONSE:** We appreciate the reviewer's questions. The central question of our study was whether there would be differences in EIT between adult and child. Adults present redistribution of ventilation from the posterior region to the anterior region with the onset of anesthesia and controlled mechanical ventilation. Our results found that the child has the same pattern of redistribution.

Therefore, in the redistribution of ventilation, there are no differences between adults and children, both present a more homogeneous ventilation (around 50%) with the onset of mechanical ventilation.

Considering that the posterior distribution in adults is around 60% and in children 54%, perhaps the magnitude in percentage of redistribution would be greater in adults, but only speculation.

**TEXT** *There was a redistribution of ventilation from the posterior to the anterior region with the onset of MV (posterior ventilation went from 54% (IC95%: 49-60%) to 49% (IC95%:44-53%) (p = 0.002) and remained stable over the whole ventilation period.(Results)*

*All text discussion*

**MANUSCRIPT LOCATION**: Section “Results”, page 11 and “Discussion”
